# EXAMINING WHETHER LIFESTYLE ACTIVITY PATTERNS PREDICT DEMENTIA INCIDENCE AMONG COMMUNITY-DWELLING OLDER ADULTS

**DOI:** 10.1093/geroni/igz038.2993

**Published:** 2019-11-08

**Authors:** Kyle Moored, Kyle Moored, Jeanine Parisi, Michelle Carlson

**Affiliations:** 1 Johns Hopkins, Baltimore, United States; 2 Johns Hopkins University, Baltimore, Maryland, United States

## Abstract

Engagement in lifestyle activities can be neuroprotective, but it remains unclear what aspects of engagement are most beneficial. Examining activity patterns may better characterize both quantitative (e.g., number) and qualitative (e.g., characteristic/motivational) differences in engagement. We used a novel, latent class analysis (LCA) to characterize subgroups with distinct activity patterns and examined whether they have differential risk of incident dementia. We compared these findings to models including standard activity frequency and variety metrics. Using the Ginkgo Evaluation of Memory Study (N=3,069), we fit Cox regressions of each activity metric on time to dementia, adjusting for intervention group and demographics. For the LCA, we derived group/class indicators for Cox regression. Variety predicted incident dementia and will be compared to LCA activity metrics in predicting risk. Activity metrics that are most protective against dementia inform intervention design. Unlike standard activity metrics, LCA may further identify subgroups with common motivations to sustain activity.

